# Optimized machine learning and artificial neural networks for NIRS-based prediction of mango internal quality

**DOI:** 10.1038/s41598-026-56804-y

**Published:** 2026-06-13

**Authors:** Kusumiyati Kusumiyati, Wawan Sutari, Unang Supratman, Agus Arip Munawar, Ine Elisa Putri, Yuda Hadiwijaya

**Affiliations:** 1 Department of Agronomy, Faculty of Agriculture, Universitas Padjadjaran, Sumedang, 45363 Jawa Barat Indonesia; 2 Department of Chemistry, Faculty of Mathematics and Natural Science, Universitas Padjadjaran, Sumedang, 45363 Jawa Barat Indonesia; 3 Department of Agricultural Engineering, Pusmeptan/PR-ITP/ARC Research Center, University Syiah Kuala, Banda Aceh, 23111 Aceh Indonesia; 4 Laboratory of Horticulture, Faculty of Agriculture, Universitas Padjadjaran, Sumedang, 45363 Jawa Barat Indonesia; 5 Doctoral Program of Agricultural Science, Faculty of Agriculture, Universitas Padjadjaran, Sumedang, 45363 Jawa Barat Indonesia; 6 Yunosato Aquaphotomics Laboratory, Hashimoto, 648-0086 Wakayama Japan

**Keywords:** Mango quality, NIR spectroscopy, Hyperparameter optimizing, Artificial neural networks, Non-destructive analysis, Chemistry, Engineering, Mathematics and computing, Plant sciences

## Abstract

A robust and updated approach for non-destructive prediction of key quality attributes in intact mangoes was developed by integrating near-infrared reflectance spectroscopy (NIRS) with advanced machine learning and artificial neural network (ANN) algorithms. In this study, NIR spectral data of 136 mango samples were collected and processed using standard normal variate (SNV) correction. Five regression methods namely partial least squares regression (PLSR), support vector machine regression (SVMR), random forest regression (RFR), extreme gradient boosting (XG-Boost), and generalized regression neural network (GRNN), were optimized and evaluated for predicting total acidity (TA) and ascorbic acid (AA). Results indicate that while traditional linear methods like PLSR achieved reasonable predictive power (RPD > 2.5), nonlinear models, especially XGBoost and GRNN, significantly outperformed PLSR, with GRNN models achieving the highest accuracy, with maximum performance reaching R^2^ up to 0.98 and RPD > 5.5 across the evaluated parameters (TA and AA). The findings demonstrate that optimized machine learning and ANN models offer robust, accurate, and practical solutions for rapid, non-invasive mango quality assessment. This integrated methodology supports advanced quality control, sorting, and breeding programs, providing substantial benefits for industry and supply chain management through rapid, reliable assessment of fruit nutritional and chemical properties.

## Introduction

Near infrared reflectance spectroscopy (NIRS) has established itself as a valuable tool in agriculture due to its rapid, non-destructive ability to assess various chemical properties of organic and inorganic materials. One of the most economically significant tropical fruits is the mango (*Mangifera indica* L.), whose internal quality characteristics are crucial to consumer acceptance and market value. In order to facilitate postharvest handling and quality control, it is imperative that fast and non-destructive methods for evaluating mango quality be developed. NIRS relies on the interaction of near infrared radiation, approximately between 780 and 2500 nm, sometimes extended to visible light for VNIR with molecular bonds in organic matter, producing unique absorbance or reflectance spectra that correlate to the chemical composition, cell structure, and physical properties of samples^1,2^.

Total acidity (TA) and ascorbic acid (AA), are among the most critical quality attributes in mango fruit, directly affecting both fruit physiology and consumer satisfaction. TA is a key determinant of flavor, contributing to the perceived freshness and palatability by balancing sweetness and tartness, and is commonly used as an indicator of maturity and postharvest quality^3,4^. Another important nutritional component of mangos is ascorbic acid, which is often used to determine the nutritional value of fruits.

Reliable and non-destructive measurement of TA and AA in mangoes is, therefore, crucial not only for maintaining fruit quality and safety throughout the supply chain but also for delivering health-promoting nutrients to consumers. Advanced analytical approach like NIRS enables rapid and accurate monitoring of these parameters and support better postharvest management and quality evaluation^5–7^.

Each biological object like fruits generates characteristic spectral signatures based on variations in reflectance or absorbance at particular wavelengths, which can be interpreted using chemometric approaches and machine learning models to predict target attributes. However, NIR spectral data are usually high-dimensional and frequently linked to relatively small sample sizes, which poses problems for model construction, such as overfitting and multicollinearity. Thus, choosing the right model and managing dimensionality are essential for creating reliable prediction models. The practical workflow typically involves acquiring raw spectra from the sample, preprocessing the spectra to reduce noise and correct for baseline shifts using techniques like standard normal variate transformation, Savitzky-Golay derivative transformations, or multiplicative scatter correction^8^. Calibration models, often built with training sets containing known reference values to relate the spectral data to the desired chemical or physical properties. This approach allows for simultaneous prediction of multiple attributes and is considered environmentally friendly since it does not require chemical reagents or destructive sample handling^9–11^.

Extracting quantitative information from NIR spectra requires multivariate calibration, or chemometrics. Regression methods such as partial least squares regression (PLSR) are widely used due to their simplicity and ability to handle collinear and high-dimensional data by projecting both predictors and responses into lower-dimensional spaces^12,13^. PLSR models have proven effective for a range of properties like acidity and sweetness in mango^14–16^, macro and micro nutrients in soils^17–21^, chemical properties in coffee beans^22–26^, with the accuracy often quantified^27,28^.

However, the relationships between spectra and target properties are sometimes nonlinear, especially in complex matrices such us intact fruit^29,30^. Nonlinear machine learning methods such as support vector machine regression (SVMR), artificial neural networks (ANN), and ensemble tree-based methods like random forest regression (RFR) and extreme gradient boosting (XGBoost) come into play^31–33^. SVMR uses kernel functions to project data into nonlinear feature spaces, allowing for more flexible modeling of complex relationships^34^. ANN mimics neural networks to extract patterns from noisy, multidimensional data and has shown superior performance in scenarios where underlying relationships are nonlinear^35^. Ensemble methods like RFR and XGBoost combine multiple decision trees to achieve high accuracy and robustness, especially when properly tuned^36^.

Recent studies demonstrate that nonlinear machine learning approaches cam improve prediction accuracy in NIRS–based fruit quality assessment^37–39^. In fruit quality assessment, such as total acidity prediction in mango, NIR combined with chemometrics and machine learning has achieved excellent accuracy. Recent research comparing PLSR, SVMR, and ANN found that ANN, with optimized principal component inputs derived from standard normal variate (SNV) processed spectra, provided the highest R² and lowest prediction errors, achieving an RPD of over 4 in prediction, well above the threshold for excellent prediction accuracy^35^. The method allows for rapid, objective grading of fruits based on internal chemical quality without damaging produce.

The integration of advanced machine learning models and ANNs has become increasingly important for maximizing the accuracy and robustness of NIRS based quality assessment in agricultural products. These sophisticated algorithms each offer unique strengths in capturing the complex, often nonlinear relationships inherent in NIR spectral data and the diverse quality attributes of agricultural samples. SVMR excels in modeling nonlinear interactions within data through its use of kernel functions, making it particularly effective for handling the variability found in natural products. RFR and XGBoost, both based on the principles of ensemble learning, use aggregations of decision trees to minimize prediction errors, enhance model stability, and reduce the risks of overfitting, which are common issues in high-dimensional spectral datasets^40,41^. Meanwhile, ANNs are uniquely equipped to model complex multidimensional patterns due to their layered structure, enabling superior performance in cases where spectral signatures are intricate and conventional statistical methods may fall short.

To realize the full predictive capabilities of these algorithms, it is essential to employ a strategic approach to model selection, choosing models based not only on their theoretical capabilities but also on their fit to specific datasets and prediction tasks. Furthermore, rigorous hyperparameter optimization is crucial to achieving top model performance. Conventional methods such as grid search are often computationally intensive. Thus, more efficient strategies like Bayesian optimization are now widely adopted^42^. These optimization approaches systematically explore the model parameter space to identify the best-performing configurations, thereby improving predictive accuracy, generalizability across different sample sets, and overall model robustness. The synergy of these machine learning innovations with well-designed NIRS workflows enables the development of reliable, rapid, and scalable solutions for non-destructive quality assessment of crops, fruits, and soils, ultimately supporting high quality agricultural production and objective quality control at scale.

Therefore, the novelty of this study lies in the comparative evaluation of multiple machine learning methods, such as optimized SVMR, RFR, XGboost and ANNs, alongside strategic model selection, rigorous hyperparameter optimization, and comprehensive spectral preprocessing in order to enhance predictive power and ensure broad applicability across varied samples and environment. The purpose of this presented work is to develop and validate accurate, update, and robust NIRS prediction models for non-destructive and simultaneous assessment of essential quality traits including TA and AA in intact mango fruits. The objective of this work is to support the development of dependable non-destructive quality evaluation systems in horticulture applications and to give a better knowledge of model performance in relation to spectral data features.

## Materials and methods

A total of 136 intact mango fruits (cv. *Kent* and *Palmer*) originating from Spain, Brazil, and Peru were procured from local markets in Göttingen, Germany. The fruits were stored at ambient temperature (approximately 25 °C) and evaluated over a storage period of approximately 13 days. At each measurement session (every two days), a subset of approximately ten mangoes were selected for both near-infrared spectral data acquisition and subsequent determination of actual TA and AA using established laboratory methods. Prior to the destructive assessment of TA and AA, NIR spectra were collected from each intact fruit. Every mango was assigned a unique sample number, after which spectral data were obtained and, immediately afterwards, the fruit underwent TA analysis in sequence.

### NIR spectral data

In this study, NIR spectral data, expressed as absorbance (Log(1/R)), were collected using a benchtop Nicolet-Antaris Method Development Sampling (MDS) system (Thermo Fisher Scientific Inc., Madison, WI, USA). Instrument operation and configuration were managed through integrated Thermo Integration^®^ and Thermo Operation^®^ software, with the specific data acquisition workflow constructed via Thermo Integration. Spectral measurements for all samples were obtained in the range of 10,000 to 4000 cm^−1^ or corresponding to 1000 to 2500 nm, utilizing an integrating sphere as the default measurement setup, and each spectrum was generated by averaging 64 co-added scans per acquisition^8,35^. The near-infrared region, which is frequently utilized in NIRS research because of its sensitivity to overtone and combination vibrations of important functional groups including O–H, C–H, and N–H, is represented by the chosen spectral range of 10,000–4000 cm^−1^ (1000–2500 nm). Water, organic acids, and other chemical components important to mango quality, such as TA and AA, are tightly linked to these functional groups. The instrument employed a white teflon block as the reference standard, which was automatically recalibrated every hour to maintain accuracy. Scanning was performed at an operating temperature maintained between 28 and 31 °C^10^. For each mango sample, spectra were captured at four distinct locations, the north edge, center, and south edge of the intact mango fruit, and the resulting measurements were averaged to represent each sample.

### AA measurement

After spectral data collection, each mango was sliced at the identical marked sites used for NIR acquisition, and the pulp was retrieved for further analysis. AA was determined first, as this compound is highly volatile and prone to rapid oxidation upon exposure to air following slicing. Quantification of AA employed a titrimetric method using 2,6-dichlorophenolindophenol (DCPIP) solution^43,44^. Five grams of mango pulp were macerated and combined with 20 ml of 5% metaphosphoric acid (Roth, Germany) in a beaker, serving to inhibit oxidation during analysis. The mixture was homogenized using an Ultra-Turrax blender (IKA T 18B, Germany) for approximately 2 min. The solution volume was adjusted to 50 ml with distilled water, then filtered using MN 6151/4 filter paper (150 mm diameter, Macherey-Nagel, Germany). A 10 ml aliquot of the filtrate was transferred into a 25 ml beaker for titration with 0.064 M DCPIP solution. AA, expressed as mg. 100 g^− 1^, was quantified based on the titration endpoint, indicated by a color change from colorless to light red^3^.

### TA measurement

Reference measurements for mango TA were performed immediately following spectral acquisition for each sample. After collecting and recording the NIR spectra, the mango was sliced at the identical marked locations corresponding to the spectral scan points, and the pulp was extracted for analysis. TA determination involved preparing a juice sample from 20 g of mango pulp, which was then diluted with up to 100 ml of distilled water^3,45^. The resulting mixture was clarified by centrifugation at 20 °C and 10,000 rpm for approximately 10 min to separate suspended solids, following the procedure described by Teerachaichayut and Ho[65. Acidity was determined via automatic titration (Titroline 96, Schott Geraete GmbH, Germany) using 0.1 N NaOH until an endpoint of pH 8.1 was reached, and reported as mg of acid per 100 g (mg.100 g^− 1^). TA values were determined using a standard titration method, measured in triplicate for each sample, and the results were averaged^4^.

### Spectral data enhancement

To ensure the development of reliable, accurate, and robust prediction models, the NIR spectra from all mango samples were subjected to SNV correction prior to analysis. Previous research has demonstrated that the application of SNV, particularly when combined with the PLSR modeling approach, significantly improves predictive performance for assessing quality traits in intact mango fruit^3,8^. The SNV technique works by minimizing the influence of light scattering effects, transforming each spectrum towards an ‘ideal’ baseline through linearization. Specifically, this correction involves normalizing each individual spectrum to have a mean of zero and a standard deviation of one, thereby improving data comparability and model stability.

### Calibration models

Calibration models for the prediction of TA and AA in mangoes were developed using the enhanced NIR spectral data. Five different regression techniques were employed: PLSR, SVMR, RFR, XGBoost, and ANN. The dataset comprising 136 mango samples was divided into two subsets: a calibration set of 103 and independent prediction set of 33 samples. The calibration set was selected to represent the full of reference values for both TA and AA.

For PLSR, the optimal number of latent variables (LVs) was determined by leave-one-out cross-validation (LOOCV) applied to the calibration set. LVs were incrementally added up to a maximum of 20, and the number yielding the minimum root mean square error of cross-validation (RMSECV) was selected. This approach ensures that the PLSR model captures the maximum relevant spectral variance without overfitting to calibration noise. To address the high dimensionality of the NIR spectral dataset approximately 6000 variables per sample relative to the limited calibration sample size *n* = 103, principal component analysis (PCA) was applied prior to training the machine learning models (SVMR, RFR, XGBoost, and GRNN). The number of principal components (PCs) retained was determined by the criterion of cumulative explained variance, keeping the minimum number of PCs that collectively accounted for at least 99% of the total spectral variance. This PC score matrix, rather than the full raw spectral array, was used as the input feature space for all machine learning models, ensuring that the effective input dimensionality was substantially smaller than the calibration sample size and thus mitigating the risk of overfitting. All analyses were performed in Python 3.10 using the following libraries: scikit-learn v1.3 (PLSRegression, SVR with GridSearchCV, RandomForestRegressor with RandomizedSearchCV, PCA), xgboost v1.7 with Bayesian hyperparameter optimization via hyperopt v0.2.7 (Tree-structured Parzen Estimator), and neupy v0.8 for GRNN implementation.

To further enhance model performance, hyperparameter optimization procedures were systematically applied to the SVMR, RFR, and XG-Boost regression algorithms. For SVMR, the penalty parameter (C), kernel width (gamma), and the epsilon parameter were optimized using a structured grid search over a predefined candidate grid: C ∈ {0.1, 1, 10, 100}, gamma ∈ {0.001, 0.01, 0.1, 1}, and epsilon ∈ {0.01, 0.05, 0.1, 0.5}. The radial basis function (RBF) kernel was selected based on its established effectiveness for nonlinear spectral regression tasks. For RFR, the primary parameters optimized included the number of trees in the forest (n_estimators ∈ {50, 100, 200, 300}), maximum tree depth (max_depth ∈ {None, 10, 20, 30}), and the minimum samples required for splitting (min_samples_split ∈ {2, 5, 10}) and leaf nodes (min_samples_leaf ∈ {1, 2, 4})^46–48^. XG-Boost model optimization focused on learning rate (eta ∈ {0.01, 0.05, 0.1, 0.2}), maximum tree depth (max_depth ∈ {3, 5, 7, 9}), subsample ratio (subsample ∈ {0.6, 0.8, 1.0}), column subsampling ratio (colsample_bytree ∈ {0.6, 0.8, 1.0}), and number of estimators (n_estimators ∈ {100, 200, 300, 500}). Due to the larger combined hyperparameter space, Bayesian optimization using a Tree-structured Parzen Estimator (TPE) was employed with 50 evaluation trials and five-fold cross-validation, targeting the lowest RMSECV. Bayesian optimization was chosen over exhaustive grid search for XG-Boost because it sequentially builds a probabilistic model of the objective function and focuses evaluations on promising regions of the parameter space, thereby achieving efficient exploration without requiring an intractable number of model fits. For GRNN, only the smoothing parameter (sigma) required tuning, which was optimized by evaluating values in the range {0.01, 0.05, 0.1, 0.2, 0.5, 1.0} using leave-one-out cross-validation on the calibration set, selecting the value that minimized RMSECV. Model performance at each configuration was assessed using cross-validation procedures targeting the lowest RMSE or highest RPD in the prediction data. Model performance at each configuration was assessed using cross-validation procedures targeting the lowest RMSE or highest RPD in the prediction set, ensuring optimal predictive accuracy and generalizability for both TA and AA prediction in mango.

The GRNN architecture follows the design proposed by Specht and consists of four functional layers. The Input layer receives the reduced-dimensionality PC score vector for each sample. The Pattern layer contains one neuron per calibration sample, each computing a radially symmetric Gaussian kernel probability density function of the form K(x, x_i_) = exp(−||x − x_i_||²/(2σ²)), where σ is the smoothing bandwidth parameter and ||x − x_i_|| is the Euclidean distance between the query input x and the *i*th training sample x_i_. The Summation layer consists of two units: a numerator summation unit that computes the weighted sum of training outputs using the kernel activations as weights, and a denominator summation unit that computes the sum of all kernel activations. The Output layer yields the final predicted value as the ratio of the numerator to the denominator, implementing the Nadaraya and Watson kernel regression estimate. Cross validation was applied throughout the model development process as follows: for PLSR, LOOCV on the calibration set was used to select the optimal number of LVs; for SVMR, five-fold cross-validation within GridSearchCV was used to evaluate each hyperparameter combination; for RFR, five-fold cross-validation within RandomizedSearchCV was used; for XGBoost, five-fold cross-validation guided each TPE trial; and for GRNN, LOOCV was used to select σ. In all cases, RMSECV was the criterion minimized during model selection. The RMSECV values reported in Table 2 correspond to the cross-validated prediction error on the calibration set under each model’s respective cross-validation scheme, providing an internal estimate of generalizability prior to final evaluation on the independent prediction set.

For both quality attributes, calibration models were constructed by associating the reference measurements (as y-variables) with the corresponding NIR spectra (as x-variables) within the calibration set. Model performance was evaluated against the prediction set using the following metrics: coefficient of determination for calibration (R^2^c) and prediction (R^2^p), root mean square error of calibration (RMSEC) and prediction (RMSEP), the difference between RMSEC and RMSEP, and the ratio of prediction to deviation (RPD), calculated as the ratio of the standard deviation of the reference values (SDref) to the RMSEP. The number of principal components or latent variables utilized in each model was also considered for optimal model selection. All spectral data corrections, PLSR and SVMR were analyzed by means of The Unscrambler X 10.3 with network Client License (CAMO, Oslo Norway), while other Machine Learning Algorithms RFR, XG-Boost and GRNN were performed using R software version 4.5.2.The R library for RFR is randomForestSRC, X-boost library using xgboost, while GRNN library is tsfgrnn.

Model accuracy was principally assessed using the RPD index: values of 1.5–1.9 indicate moderate quantitative prediction potential, 2.0–2.5 denote sufficient performance for practical use, and RPD > 2.5 reflects good to excellent predictive accuracy^5,7,8,49,50^. Additionally, for PLSR, the regression coefficient curve was used to evaluate the importance of different NIR wavelengths in predicting TA and AA levels in mango, thereby assisting in the identification of relevant spectral regions for each quality attribute.

## Results and discussion

### Intact mango fruit’s NIR spectral characteristics

**Fig. 1:**
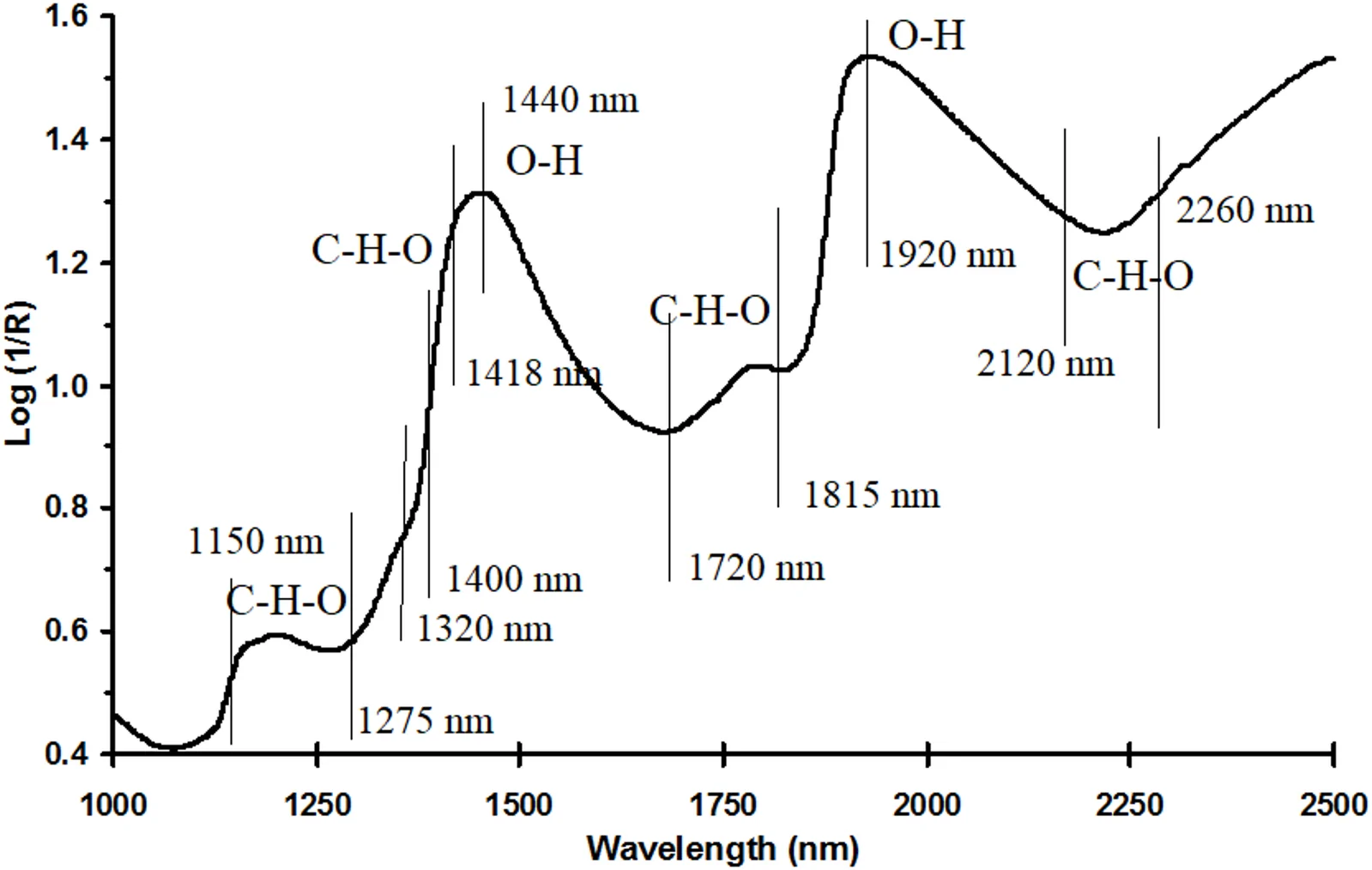
Typical near infrared spectra of intact mango in form of absorbance (log (1/R)) in wavelength range from 1000 to 2500 nm.

The NIR spectral features of intact mango fruit in the NIR wavelength region is presented in Fig. 1. It shows a holistic view of the mango’s chemical composition and physical structure through its absorbance features. Major absorption bands can be observed at characteristic wavelengths, primarily due to overtone and combination vibrations of O–H, C–H, and N–H chemical bonds, which are abundantly present in water, sugars, acids, and other organic constituents of mango pulp and peel.

The 1000–1400 nm region is predominantly governed by the first overtone of O–H stretching vibrations. This spectral interval responds strongly to changes in the fruit’s water content, a major constituent of mango tissue^9,51^. Variability in absorbance within this area is thus closely linked to the hydration status and juiciness of different mango samples. In practical terms, any differences in ripeness, internal structure, or storage conditions that affect water distribution are captured sensitively in this segment of the spectrum, making it essential for the accurate non-destructive evaluation of mango freshness and quality.

On the other hand, between 1400 and 1900 nm region of the NIR spectrum is characterized by robust absorbance features arising from both O–H and C–H bond overtones and combination bands^52,53^. This range is especially informative for organic molecules such as sugars like glucose, fructose, sucrose and organic acids, which directly govern the fruit’s sweetness and total acidity. Therefore, absorbance patterns in this zone provide crucial spectral fingerprints tied to the mango’s flavor profile and nutritional value, including parameters such as ascorbic acid (AA)^54^. The strong signals from water superimposed with organic components support the construction of chemometric models targeting total acidity and ascorbic acid, enabling their prediction through advanced multivariate regression and machine learning approaches.

Furthermore, the 1900–2500 nm region encompasses combination vibrations of O–H, N–H, and C–H bonds and thus encodes the spectral signatures of water, proteins, and more complex carbohydrates^55,56^. Peaks and valleys appearing in this longer-wavelength region reflect not just the gross contents of water and sugars but also more subtle compositional details among mango samples, such as differences in protein content or the nature of polysaccharides in the pulp and skin. Models that incorporate this region are better equipped to distinguish between samples with small differences in internal composition, significantly enhancing the robustness and generalizability of NIRS-based predictions for quality attributes. These spectral assignments are not only consistent with the physical-chemical bases of NIR absorption but are also supported by findings in published NIR studies of mango and other fruits, underlining the method’s effectiveness for rapid, simultaneous quality trait estimation.

The spectra feature displays not only the key absorbance of intact mango fruit, but also demonstrate a consistently clear baseline and strong signal to noise characteristics across the entire measured wavelength interval^57^. Such spectral quality is crucial during model construction, as it reduces the influence of unwanted noise, background variations, and other measurement distortions that could otherwise compromise the true chemical signals needed for accurate regression.

When spectral data are this stable and reproducible, even subtle but significant differences among samples, for example, changes in chemical properties, moisture levels, or ripeness, remain distinguishable. This level of reliability is especially important for advanced chemometric and machine learning analyses, since these predictive techniques are designed to uncover and model nuanced spectral patterns linked with properties like TA and AA content.

Due to this high standard of spectral integrity, the various multivariate and nonlinear modeling approaches used in this work (PLSR, SVMR, RFR, XGBoost, ANN) can fully capitalize on all available data. These methods can detect both obvious and subtle spectral variations, allowing the models to achieve high predictive accuracy and robustness. This ensures that the models remain resilient to overfitting and are able to generate quantitative predictions for external prediction datasets, strengthening both the scientific reliability and practical value of the methodology.

The descriptive statistics for both the calibration and prediction sets are presented in Table 1. They provide a strong foundation for developing and validating robust NIRS models to predict mango quality attributes. The calibration dataset, comprising 103 samples, exhibits a wide distribution of TA, with values ranging from 111.67 to 993.83 mg.100 g^− 1^, yielding a mean of 462.88 mg.100 g^− 1^ and a substantial overall range of 882.17 mg.100 g^− 1^. Similarly, AA content in this set spans from 18.49 to 73.19 mg.100 g^− 1^, with a mean of 42.31 mg.100 g^− 1^ and a total range of 54.70 mg.100 g^− 1^. The high standard deviations and ranges in these values indicate that the calibration set captures extensive natural variation in key chemical quality indicators, covering a broad spectrum of ripening stages, cultivar differences, and potential postharvest handling effects.

**Table 1:** Descriptive statistics of actual TA and AA measurements from calibration and prediction dataset.

Statistical indicator	Calibration dataset (*n* = 103)		Prediction dataset (*n* = 33)	
	TA (mg.100 g^− 1^)	AA (mg.100 g^− 1^)	TA (mg.100 g^− 1^)	AA (mg.100 g^− 1^)
Mean	462.88	42.31	491.65	48.40
Max	993.83	73.19	766.50	71.62
Min	111.67	18.49	245.10	24.11
Range	882.17	54.70	521.40	47.51
Std. Dev.	177.26	14.24	131.46	12.96
Variance	31420.48	202.67	17282.79	167.86
RMS	495.35	44.62	508.41	50.05
Skewness	0.31	0.34	0.25	− 0.08
Kurtosis	0.21	− 0.84	− 0.59	− 0.98
Median	438.90	40.40	451.60	49.38

The independent prediction set contains 33 samples and maintains a similarly diverse range. For TA, the values stretch from 245.10 to 766.50 mg.100 g^− 1^ (mean: 491.65 mg.100 g^− 1^, range: 521.40 mg.100 g^− 1^), while AA concentrations vary from 24.11 to 71.62 mg.100 g^− 1^ (mean: 48.40 mg.100 g^− 1^, range: 47.51 mg.100 g^− 1^). These ranges overlap with but are not entirely contained within the calibration set, which is ideal for testing a model’s true predictive ability on unseen data and for assessing generalizability.

This careful design, capturing substantial heterogeneity in TA and AA across both datasets, is essential for NIRS regression model training. It ensures the models are exposed to and learn from a diversity of ground truth values, thereby minimizing bias and enhancing robustness when deployed on real-world mango batches. The comprehensive coverage also supports the development of models that remain discriminative and accurate whether predicting the acidity and AA content in mangoes from different origins, harvest times, or market conditions.

### NIRS models for TA and AA prediction

Table 2 compares the performance of five NIRS-based models PLSR, RFR, SVMR, XGBoost, and ANN, in predicting TA in intact mangoes, using both calibration and external prediction datasets. The NIR spectra displayed unique absorption characteristics associated with O–H, C–H, and N–H bonds. Differences in internal composition are reflected in variations in spectral intensity among samples, which are important for predicting AA and total acidity.

The results for the PLSR model indicate a satisfactory ability to explain the variance in both calibration (R^2^ = 0.91) and prediction (R^2^ = 0.83), reflecting that the linear model captures the main trends in the relationship between spectral data and total acidity in mangoes. However, the RMSEP value of 61.42 mg.100 g^− 1^ and an RPD of 2.14 as presented in Fig. 2 show that, despite overall reasonable predictive capacity, the model’s practical accuracy is moderate and may be limited for routine commercial or high-precision research purposes.

**Table 2:** Calibration and prediction performances of machine learning and ANN methods for total acidity determination.

Parameters	NIRS models	Statistical parameter						
		Calibration			Prediction			
		R_c_^2^	RMSEC (mg·100 g⁻¹)	RMSECV (mg·100 g⁻¹)	R_p_^2^	RMSEP (mg·100 g⁻¹)	RPD	RER
TA	PLSR	0.91	42.62	58.53	0.83	61.42	2.14	8.49
	RFR	0.94	38.24	45.28	0.91	49.67	2.65	10.50
	SVMR	0.94	37.05	44.16	0.92	47.22	2.78	11.04
	XG-boost	0.97	29.34	33.68	0.96	36.71	3.58	14.20
	ANN	0.99	20.57	24.62	0.98	27.58	4.77	18.91
AA	PLSR	0.90	3.62	4.37	0.88	4.68	2.77	10.15
	RFR	0.94	2.88	3.24	0.91	3.39	3.82	14.01
	SVMR	0.95	2.67	3.19	092	3.24	4.00	14.66
	XG-boost	0.97	2.18	2.47	0.95	2.55	5.08	18.63
	ANN	0.98	1.92	2.11	097	2.34	5.54	20.30

This moderate performance is consistent with several previous studies on NIRS-based fruit quality assessment, where PLSR provided an effective baseline for quantitative predictions, particularly when the underlying data relationships are approximately linear or the measured trait has a narrow variability range^58,59^. However, when the calibration sample set spans a broad chemical diversity, as with the present study’s mango collections, the limitations of PLSR’s linear modeling become more pronounced. To be specific, PLSR can struggle to capture the full complexity of interactions in intact biological systems, where spectral responses are often influenced by nonlinear effects such as overlapping absorption features, light scattering, cellular structural differences, and water–solute matrix interactions.

Fig. 2TA (**a**, **c**, **e**, **g**, **i**) and AA (**b**, **d**, **f**, **h**, **j**) prediction performance. Calibration (black dot) and prediction (green triangle) for TA models. Model for calibration (orange dot) and prediction (green triangle) for AA models.

**Figure:**
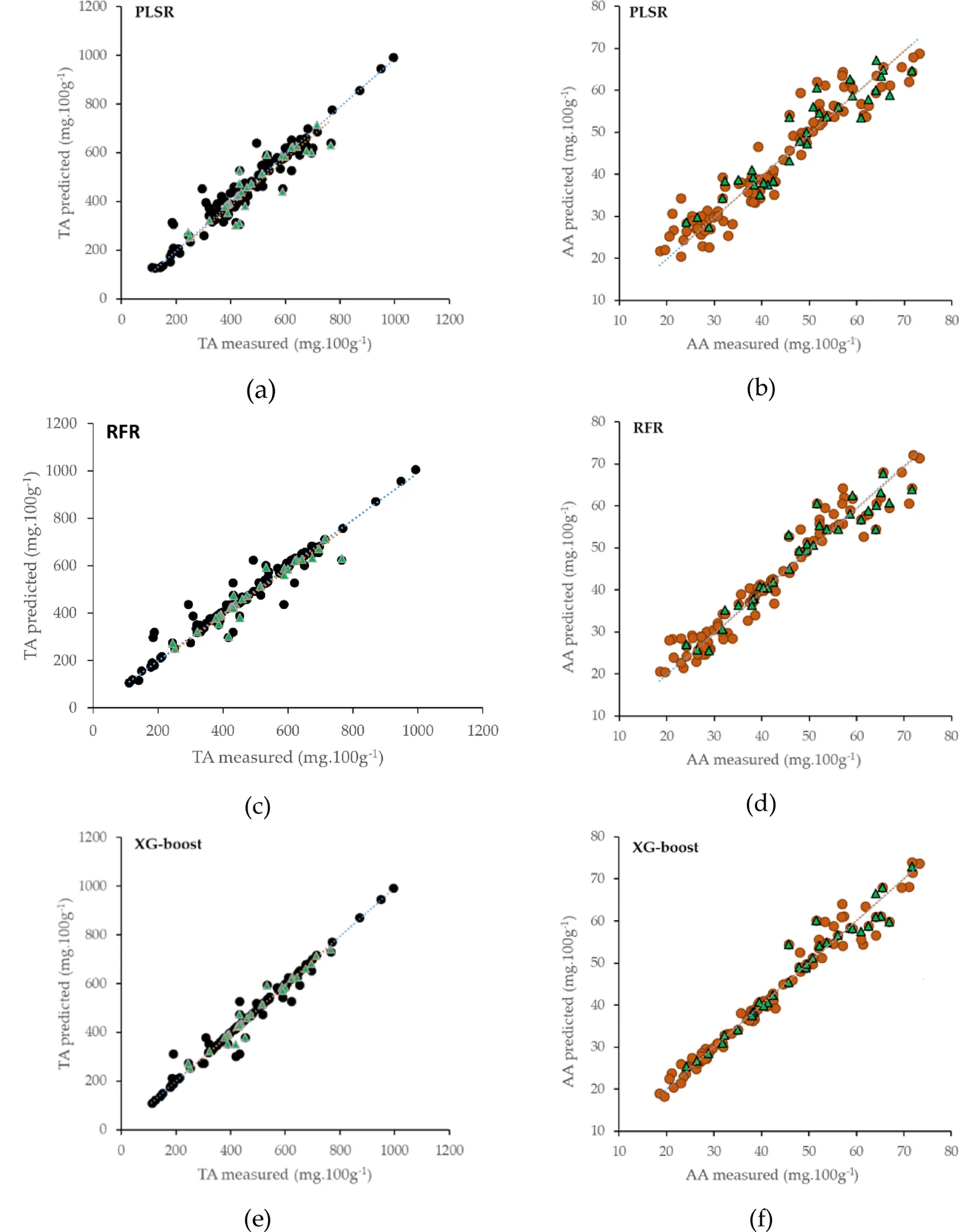

**Figure:**
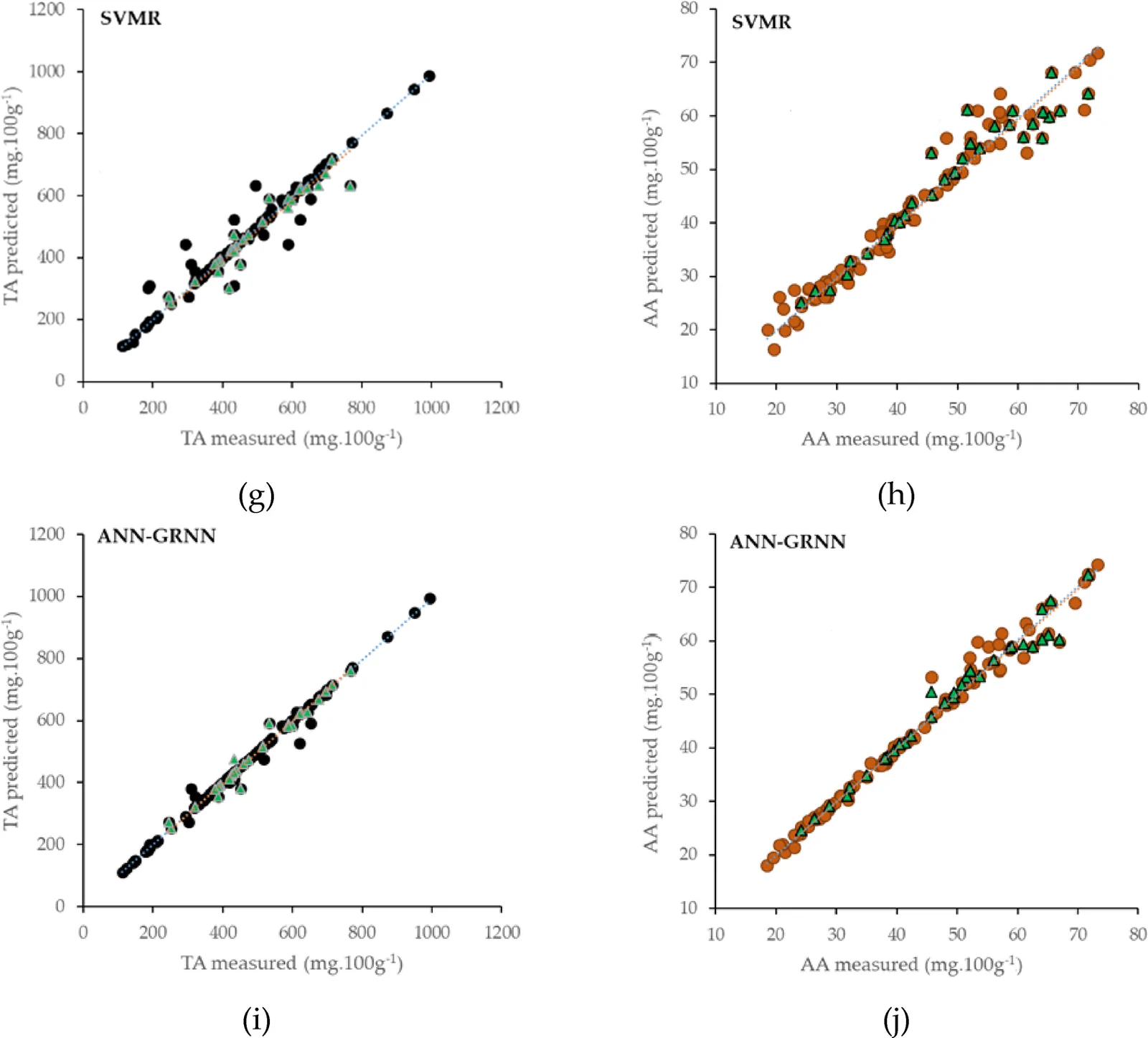


Multiple literature sources have documented this phenomenon. For example,  Hayati et al.^60^ found that while PLSR could yield RPD values between 1.5 and 3.0 for mango and other fruit quality parameters, more advanced machine learning techniques, particularly those accommodating nonlinear mapping, resulted in higher accuracy and superior generalizability. Similar findings are noted in NIR studies of citrus, berries, and apples, where machine learning algorithms consistently outperform PLSR for attributes related to acidity, AA, and other internal qualities due to their ability to leverage the detailed, often non-additive patterns within NIR data.

Although PLSR remains a valuable tool in the NIR chemometrics for its interpretability, computational efficiency, and suitability for quick, first pass screening, its moderate error and RPD values in this context highlight the need for nonlinear, data-driven models when predicting complex compositional attributes in heterogeneous fruit datasets. The trend observed here matches the growing consensus in the field, advocating for a transition toward optimized machine learning and ensemble techniques for state of the art fruit quality assessments^61^.

The performance of RFR and SVMR models in this study as shown in Fig. 2 reveals substantial gains over traditional linear approaches like PLSR. Both models produce high coefficients of determination for prediction (R^2^ = 0.91 for RFR and R^2^ = 0.92 for SVMR), indicating that they capture a larger proportion of the variance in total acidity from the NIR spectra of mangoes than PLSR does. Furthermore, the reduction in RMSEP values (49.67 mg.100 g^− 1^ for RFR and 47.22 mg.100 g^− 1^ for SVMR) underscores their effectiveness at minimizing prediction errors on unseen samples.

A critical metric for evaluating model utility in practice, the RPD, is 2.65 for RFR and 2.78 for SVMR. Both exceed the threshold of 2.5, which is generally accepted as an indicator of models that are robust and reliable for practical, real-world screening or grading applications. This improvement is underpinned by the intrinsic strengths of these methods. RFR, as an ensemble of decision trees, can account for complex, nonlinear associations inherent in biological spectral data, mitigating the impact of noise and overfitting. Similarly, SVMR leverages kernel-based mapping to resolve nonlinearities, offering nuanced calibration across diverse sample matrices.

These findings are consistently echoed in NIRS literature. Studies by Hayati et al.^60^ and related fruit quality research regularly report superior performance for ensemble tree models and SVMR over linear regression, particularly when dealing with heterogeneous sample sets or spectra reflecting nonlinear sample–matrix effects. In summary, the adoption of RFR and SVMR in mango NIR modeling provides a tangible step forward in achieving more accurate and generalizable predictions of quality traits compared to classical chemometric approaches.

The results obtained from the XG-Boost model, with a high prediction R^2^ = 0.96, low RMSEP of 36.71 mg.100 g^− 1^, and an RPD value of 3.58, demonstrate a significant leap in predictive reliability and robustness compared to both linear and standard nonlinear models. XG-Boost’s architecture, based on boosted decision trees, is highly effective at handling large, high dimensional NIR spectral datasets. It excels at selecting relevant spectral features while filtering out noise and irrelevant variation, leading to minimized generalization errors even across independent prediction sets.

The strong performance of XG-Boost in this presented work (Fig. 2) is well supported by recent publications, which highlight its superiority for complex regression tasks in fruit quality assessment, especially when spectra display nonlinearities or intercorrelated features. Research in NIRS applications to fruit sugars, acidity, and internal defects consistently reports that boosting algorithms optimize both bias and variance^7–9,49^, often outperforming simpler ensembles or single-model approaches for accuracy and reliability in practical scenarios. Furthermore, XGBoost’s gradient boosting mechanism enables efficient learning from diverse sample sets, increasing the model’s adaptability to the broad range of chemical and physical variation observed in real commercial mango batches. With an RPD well above 3.0, this model qualifies as highly robust according to accepted NIR chemometric standards, making it fit for routine high-precision grading and non-destructive quality control in industry settings.

In this study, the ANN approach implemented for predicting TA in mangoes is specifically the Generalized Regression Neural Network (GRNN), a type of probabilistic neural network renowned for its effectiveness in nonlinear regression tasks involving high-dimensional and noisy datasets like NIR spectra. Unlike traditional multilayer perceptrons, GRNN operates using a kernel regression paradigm, which allows it to model highly nonlinear and multidimensional relationships between the spectral inputs and chemical quality responses without the need for extensive parameter tuning or iterative backpropagation. The GRNN model delivered exceptional predictive performance as shown in Fig. 2. Its prediction set coefficient of determination (R^2^ = 0.98) indicates nearly perfect capture of the variance in total acidity, its RMSEP (27.58 mg.100 g^− 1^) demonstrates minimal error between predicted and reference values, and its RPD (4.77) signals excellent robustness, far surpassing the threshold (RPD > 2.5) typically associated with models suitable for precise, real-world quality applications. Such high metrics are rarely achieved by linear or shallow nonlinear techniques, illustrating how GRNN excels at learning even subtle, non-obvious patterns embedded in complex fruit NIR spectra.

The ANN-GRNN typically converges very quickly because it is not iterative, but instead uses non-parametric, instance-based learning, making it attractive for large datasets and real-time deployment. Thanks to its probabilistic smoothing-based approach, GRNN generalizes well to unseen data and is inherently less sensitive to outliers or random noise than standard neural networks or regression methods. Moreover, GRNN given sufficient data and appropriate kernel parameterization, can approximate any continuous function from spectra to target property, thus capturing nonlinear, multi-variable dependencies that frequently arise in agricultural NIR measurements^62,63^.

Advanced machine learning algorithms introduce more advanced architectures, such as boosted ensembles and neural networks, they can capture increasingly nuanced relationships between spectral data and fruit acidity. This results in fewer prediction errors and better adaptation to unseen, heterogeneous mango samples. The sizable performance advantage of ANN (GRNN) arises from its multilayer structure, which equips it to unravel intricate, nonlinear, and multi-dimensional associations between NIR spectra and chemical properties. This architectural flexibility means ANN can generalize across wide-ranging fruit batches and adapt to changing production or growing conditions.

Multiple studies on fruit and vegetable NIRS, including quality determination in mango, apple, citrus, and berries, report that GRNN or similar neural network algorithms consistently outperform not only linear chemometric approaches like PLSR but often also advanced ensemble algorithms such as RFR or XGBoost, particularly when the datasets are heterogeneous or exhibit complex, non-additive effects. These advantages have positioned GRNN as a preferred choice for robust, high-precision, non-destructive quality evaluation tasks. The results here reinforce the consensus that GRNN’s unique architecture and probabilistic learning make it exceptionally well suited to NIRS-based fruit quality assessment, achieving both high accuracy and generalizability, essential for commercial mango sorting, grading, and breeding applications.

Recent research has consistently demonstrated that ANNs and advanced machine learning models significantly outperform traditional linear chemometric approaches like PLSR when it comes to predicting quality traits such as acidity in mangoes and other fruits using NIR spectroscopy data^34,64^. The ANN models achieved the best results in terms of both RPD and RMSE for TA prediction in mangoes, even surpassing the results of SVMR and PLSR under equivalent data conditions. This superiority is attributed to ANNs’ ability to model highly nonlinear and multidimensional relationships within complex biological spectral datasets.

While Hayati et al.^60^ achieved impressively high RPD values of up to 6.77 for some quality traits by applying very carefully tuned preprocessing and optimization workflows to PLSR, their findings highlight an important caveat: such strong results from linear models are only attainable with considerable manual intervention and are typically less robust across changing sample sets or new environments. In contrast, machine learning and neural network approaches maintain high predictive accuracy and robustness with less dependence on intensive pre-processing or sample-specific recalibration, making them more scalable and practical for real-world applications.

Moreover, the trend toward deep learning and ensemble algorithms, such as XGBoost and convolutional neural networks (CNNs), is growing in NIRS-based fruit quality analytics^63^. These models are increasingly recognized for their ability to automatically extract and combine nonlinear features, handle high-dimensional data, and deliver reliable predictions across diverse and variable datasets. Their effectiveness is especially notable for tasks requiring generalizability such as grading, sorting, or quality assurance in commercial fruit processing, where classic PLSR often falls short due to its linear assumptions and lower resilience to data heterogeneity.

Beside for TA prediction, this work also reported the used of optimized machine learning and ANNs for AA. The comparative model performances for AA prediction closely mirrored those observed for 3TA, with a clear trend of increasing predictive accuracy as model complexity and nonlinearity increased. As shown in Table 2, the baseline PLSR model achieved good results, with an R^2^ in prediction of 0.88, RMSEP of 4.68 mg.100 g^− 1^, and an RPD of 2.77, exceeding the threshold (RPD > 2.5) for practical quantitative use. This demonstrates that even linear models are reasonably effective when enhanced spectral data are available and the calibration set is sufficiently diverse. Nevertheless, substantial improvements were observed as more advanced models were applied. The PLSR model for prediction in mango demonstrates good quantitative capability, as reflected in a prediction. The main advantages of the PLSR approach are its simplicity, ease of model interpretation, and relatively low computational demands, which make it attractive for rapid or preliminary assessments, especially in resource-limited settings. However, the performance of PLSR (Fig. 2) is surpassed by more advanced machine learning approaches, as observed in Table 2, which achieve higher R^2^, lower prediction errors, and superior RPD values. PLSR is constrained by its linear assumptions, it cannot fully capture the nonlinear and intricate relationships present in NIR spectra of mango, especially when the datasets are heterogeneous, and the samples present broad natural variability. This limitation can result in residual error variance that impacts the accuracy and robustness of predictions when applied to real-world mangoes from different origins, ripening stages, or handling conditions.

The RFR model demonstrated a significant enhancement in AA prediction accuracy when compared to the baseline PLSR approach. Specifically, RFR achieved a prediction R^2^ of 0.91, RMSEP of 3.39 mg.100 g⁻¹, and a robust RPD of 3.82 on the independent prediction dataset as presented in Fig. 2. These results indicate superior predictive performance and model robustness, surpassing the minimum RPD threshold for reliable quantitative analysis.

RFR’s strength lies in its ensemble architecture, which combines predictions from multiple decision trees. This design enables the model to efficiently manage high-dimensional and complex NIR spectral data and effectively model nonlinear relationships between spectral features and AA concentration. Additionally, RFR is less sensitive to the influence of measurement noise, sample outliers, and intrinsic fruit variability, minimizing the risk of overfitting, a common challenge in spectral-based predictions.

This high level of robustness means that the RFR model is well suited for application in routine mango quality control scenarios, even where there is considerable variability in fruit origin, batch, or handling conditions. By providing more consistent and reliable predictions across diverse sample sets, RFR significantly enhances the efficiency and accuracy of non-destructive AA assessment in practical, real-world settings.

Moreover, the SVMR model for the prediction of AA in intact mangoes fruits demonstrates a high degree of effectiveness, leveraging the strengths of advanced nonlinear regression for complex NIR spectral data. In the presented study, SVMR achieved a prediction set R^2^ of 0.92, with an RMSEP of 3.24 mg.100 g⁻¹ and an RPD of 4.00 as shown in Fig. 2. These statistical indicators clearly position SVMR as a top performer among conventional and ensemble learning models, confirming its reliability for quantitative NIRS-based AA analyses.

A major advantage of SVMR lies in its use of kernel functions, enabling the mapping of original spectral data into high-dimensional, nonlinear feature spaces. This fundamental property allows the algorithm to unravel and model intricate relationships that exist between the NIR spectra and target chemical components such as AA, relationships that are often masked or irretrievable by linear methods. The result is a substantial reduction in prediction error: as shown by the low RMSEP, SVMR consistently produced accurate AA estimations across mangoes with varied ripening stages, origins, and postharvest conditions. The high RPD value, exceeding the threshold of 3 considered robust for NIRS prediction, indicates that model predictions have low residual errors and are closely aligned with the laboratory reference data across diverse environmental and biological variability.

The superiority of SVMR is especially apparent when compared to both linear and standard ensemble techniques. Unlike PLSR, which is hampered by its linearity, and RFR, which relies on aggregated decision boundaries, SVMR offers a nuanced calibration approach that excels in scenarios marked by spectral nonlinearities, overlapping absorption bands, or multidimensional sample effects,a common occurrence in agricultural applications. This adaptability proves critical for mango, where the spectral signature can be influenced by cultivar differences, internal tissue heterogeneity, and storage environments.

In practical terms, the high accuracy and robustness of SVMR translate to tangible improvements for non-destructive AA assessment throughout the mango supply chain. This includes routine commercial grading, stringent sorting procedures, and breeding programs targeting enhanced nutritional qualities. The model’s performance assures stakeholders that NIRS-based SVMR models are both reliable and transferable: predictions retain their validity even when deployed across batches with new sources, handling histories, or physiological states.

The XG-Boost model represented a significant improvement in AA prediction from NIR spectra, achieving a prediction set R^2^ of 0.95, an RMSEP of just 2.55 mg.100 g⁻¹, and an outstanding RPD of 5.08 as presented in Fig. 2. These performance metrics showed XG-Boost’s exceptional precision, robust generalizability, and superior resilience when applied to heterogeneous and complex mango datasets. Notably, the RPD value far exceeds the threshold for excellent prediction, positioning XG-Boost among the highest-performing models in this comparative study.

The underlying advantages of XG-Boost originate from its boosting ensemble architecture. This approach incrementally builds an ensemble of decision trees, each of which is focused on correcting the residuals and mispredictions of its predecessors. By doing so, XG-Boost is able to extract subtle, nonlinear, and high-dimensional spectral patterns that would be missed by traditional linear or even single-tree models. This enables the system to faithfully capture the intricate relationships between the NIR spectra and ascorbic acid concentration, accounting for variations introduced by mango cultivar, ripening stage, and postharvest handling.

Another critical strength of XG-Boost lies in its automatic hyperparameter optimization and intrinsic feature selection. The algorithm efficiently navigates the trade-off between bias and variance, minimizing overfitting even as it adapts to new datasets. Its ability to auto-select the most informative wavelengths from multitudinous spectral features means that the model remains robust to noise, irrelevant variation, and measurement artifacts, common challenges in agricultural NIRS workflows. Practically, these properties make XG-Boost ideal for high-throughput, real-world mango grading and quality assurance, where many samples with substantial variance must be classified rapidly and accurately. The model’s adaptability ensures that it will maintain high performance when presented with external prediction sets, demonstrating reliability and scalability for broader industrial deployment.

For these reasons, XG-Boost stands out as a technology of choice in state-of-the-art non-destructive fruit quality analytics, enabling mango producers, breeders, and supply chains to achieve excellent, reproducible, and actionable AA quantification from NIRS measurements.

Furthermore, as also found in TA prediction, the ANN-GRNN, emerged as the top performer AA prediction in intact mangoes using NIRS data. In prediction, the GRNN achieved a prediction set R^2^ of 0.97, an RMSEP of just 2.34 mg.100 g⁻¹, and a remarkable RPD of 5.54 as presented in Fig. 2. These represent the highest accuracy and reliability among all evaluated models.

The architecture of GRNN gives it several distinct advantages. It uses a non-iterative, instance-based learning approach that employs probabilistic kernel regression. This allows GRNN to robustly model highly nonlinear and multidimensional relationships without overfitting, making it highly resistant to noise and insensitive to the existence of outliers or random fluctuations in spectral data. Because it does not rely on iterative backpropagation or complex optimization, the GRNN is well suited for real-time, high-throughput applications where large numbers of samples must be analyzed rapidly, such as during industrial grading on packing lines or in breeding selection trials.

The exceptionally high RPD value (5.54), which is well above the excellent prediction benchmark of 3, attests to the GRNN’s outstanding model robustness and predictive power. This means that the ANN-GRNN model is not only statistically strong in terms of correlation and low prediction error, but is also practically fit for immediate use in commercial quality grading, product sorting, and mango breeding programs focused on AA content. It enables growers, exporters, and food industries to nondestructively assess nutritional quality quickly, precisely, and objectively, supporting better consumer health and value-based supply chain decisions.

Deploying modern chemometric and machine learning models for NIRS-based AA prediction in mangoes brings substantial and practical benefits across screening, quality control, and breeding scenarios. Figure 2 visually supports these practical implications by comparing the prediction RMSEP and RPD values across all evaluated algorithms.

PLSR, while statistically sufficient for initial screening or rapid assessments, demonstrates higher RMSEP and modest RPD. Its linear design restricts its ability to adapt to complex or highly varied field and market samples, making it best used for low-variability, primary sorting where detailed accuracy is less crucial. On the other hand, RFR and SVMR represent a significant leap in predictive reliability. Their RMSEP values are notably lower than PLSR, accompanied by RPD values above 3, which signals excellent robustness for operational deployment. By effectively handling both biological and spectroscopic variation, these models are suited for consistent, real-world use such as in fresh produce grading, postharvest inspection, and farmer decision support where diverse origins, ripeness, and handling cannot be controlled.

XGBoost excels with an even lower RMSEP and an RPD well over 5, as illustrated in Fig. 2. This model’s performance makes it ideal where a blend of top-tier precision and processing speed is important, for example, in automated sorting lines that must classify many fruits per minute or in breeding programs seeking subtle improvements in nutritional traits. Its built-in feature selection streamlines complex NIRS data, allowing for rapid, robust predictions even as new mango varieties or seasons are encountered.

The ANN-GRNN demonstrates the lowest RMSEP and the highest RPD. This model’s probabilistic, non-iterative nature makes it remarkably insensitive to noise, resilient to new sample types, and ideal for seamless industrial-scale quality grading, research phenotyping, or breeding programs aiming for the very highest confidence in AA assessment. Its near-instantaneous predictions support integration with automated hardware and real-time sorting systems.

## Conclusion

The results of this study demonstrate that integrating advanced machine learning algorithms and ANN with NIRS can provide accurate and robust non-destructive prediction of essential quality attributes in intact mangoes, specifically TA and AA. Compared to traditional linear chemometric approaches such as PLSR, nonlinear models including RFR, SVMR, XG-Boost, and especially ANN-GRNN generally yielded higher R², lower RMSEP, and substantially improved RPD index for both TA and AA prediction. The ANN-GRNN model, in particular, achieved the best overall performance, exceeding RPD values of 4.7 for TA and 5.5 for AA, suggesting strong predictive capability under the conditions of this research. The findings underscore the critical importance of employing nonlinear, data-driven modeling strategies, robust spectral preprocessing, and comprehensive prediction to fully exploit the information contained in NIR spectra of complex biological samples. The reliability of the results is supported by the reality that the models were created and assessed using a dataset of about 133 samples, including an independent prediction set. To improve model generalizability, additional validation across cultivars, growth conditions, and larger-scale datasets is still suggested. Ultimately, the integrated NIRS-machine learning framework established in this work enables rapid, objective, and scalable assessment of internal fruit quality, supporting commercial mango sorting, grading, breeding selection, and nutritional assurance throughout the supply chain. This represents a substantial advancement in the adoption of state-of-the-art, non-invasive quality analytics in agriculture, with direct relevance to broader fruit and crop quality monitoring programs.

## Data Availability

The data that support the findings of this study are available from the corresponding author upon reasonable request.

## References

[1] Pasquini, C. Near infrared spectroscopy: a mature analytical technique with new perspectives–a review. *Anal. Chim. Acta*. **1026**, 8–36 (2018). 29852997 10.1016/j.aca.2018.04.004

[2] Hadiwijaya, Y. & Putri, I. E. Spectroscopy in food and agriculture: a critical review of applications and adoption challenges. *Food Humanity*. **5**, 100800 (2025).

[3] Munawar, A. A., von Hörsten, D., Wegener, J. K., Pawelzik, E. & Mörlein, D. Rapid and non-destructive prediction of mango quality attributes using Fourier transform near infrared spectroscopy and chemometrics. *Eng. Agric. Environ. Food*. **9**, 208–215 (2016).

[4] Munawar, A. A., Zulfahrizal, Meilina, H. & Pawelzik, E. Near infrared spectroscopy as a fast and non-destructive technique for total acidity prediction of intact mango: comparison among regression approaches. *Comput. Electron. Agric.* **193**, (2022).

[5] Sun, Y. et al. Using VIS-NIR spectroscopy and multi-omics analysis to compare mango anthracnose under natural and inoculated conditions. *Food Res. Int.* **211**, 116492 (2025). 40356146 10.1016/j.foodres.2025.116492

[6] Guru, D. S. & Nandini, D. Mango Internal Defect Detection using NIR Spectroscopy Data. *Procedia Comput. Sci.* **258**, 947–960 (2025).

[7] Castillo-Girones, S. et al. Artificial neural networks in agriculture, the core of artificial intelligence: What, When, and Why. *Comput. Electron. Agric.* **230**, (2025).

[8] Munawar, A. A., Hizir, Erika, C. & Pawelzik, E. Fast and simultaneous prediction of inner quality parameters on intact mangos by near infrared spectroscopy: Impact of spectra pre-processing on prediction accuracy. *Future Foods*. **10**, 100463 (2024).

[9] O’Brien, C. et al. Non-destructive methods for mango ripening prediction: Visible and near-infrared spectroscopy (visNIRS) and laser Doppler vibrometry (LDV). *Postharvest Biol. Technol.* **212**, (2024).

[10] Chuquimarca, L. E., Vintimilla, B. X. & Velastin, S. A. A review of external quality inspection for fruit grading using CNN models. *Artif. Intell. Agric.* **14**, 1–20 (2024).

[11] Kusumiyati, K., Putri, I. E., Hadiwijaya, Y. & Munawar, A. A. Transforming gotu kola (Centella asiatica L.) chemical properties inspection through advanced spectroscopy technology. *Case Stud. Chem. Environ. Engineering* **10**, (2024).

[12] Mahanti, N. K. et al. Emerging non-destructive imaging techniques for fruit damage detection: Image processing and analysis. *Trends Food Sci. Technol.* **120**, 418–438 (2022).

[13] Kusumiyati, K. et al. Quality assurance of total carotenoids and quercetin in marigold flowers (Tagetes erecta L.) as edible flowers. *Int. J. Food Sci.* (2025). (2025). 10.1155/ijfo/3277288 PMC11753848 39845693

[14] Munawar, A. A., Zulfahrizal, Meilina, H. & Pawelzik, E. Near infrared spectroscopy as a fast and non-destructive technique for total acidity prediction of intact mango: Comparison among regression approaches. *Comput. Electron. Agric.* **193**, 106657 (2022).

[15] Raghavendra, A., Guru, D. S. & Rao, M. K. Mango internal defect detection based on optimal wavelength selection method using NIR spectroscopy. *Artif. Intell. Agric.* **5**, 43–51 (2021).

[16] Li, L., Jang, X., Li, B. & Liu, Y. Wavelength selection method for near-infrared spectroscopy based on standard-sample calibration transfer of mango and apple. *Comput. Electron. Agric.* **190**, 106448 (2021).

[17] Du, X. et al. Quantitative analysis of soil potassium by near-infrared (NIR) spectroscopy combined with a three-step progressive hybrid variable selection strategy. *Spectrochim Acta Mol. Biomol. Spectrosc.* **324**, 124998 (2025). 10.1016/j.saa.2024.124998 39178690

[18] Huang, J., Mao, Z., Xiao, D., Fu, Y. & Li, Z. A novel quantitative detection method for soil organic matter content based on visible to near-infrared spectroscopy. *Soil. Tillage Res.* **244**, 106247 (2024).

[19] Dai, L. et al. In-situ prediction of soil organic carbon contents in wheat-rice rotation fields via visible near-infrared spectroscopy. *Soil. Environ. Health*. **2**, 100113 (2024).

[20] Wei, J. et al. Correlation analysis between active components of Cornus officinalis and inorganic elements in rhizosphere soil and rapid analysis of origin quality by near-infrared spectroscopy combined with machine learning. *Ind. Crops Prod.* **210**, 118101 (2024).

[21] Xu, S., Zhao, Y. & Wang, Y. Optimizing machine learning models for predicting soil pH and total P in intact soil profiles with visible and near-infrared reflectance (VNIR) spectroscopy. *Comput. Electron. Agric.* **218**, 108643 (2024).

[22] Mutz, Y. S. et al. Feasibility of NIR spectroscopy coupled with chemometrics for classification of Brazilian specialty coffee. *Food Control*. **149**, 109696 (2023).

[23] Zhang, Q. et al. Quantitative determination of TVB-N content for different types of refrigerated grass carp fillets using near-infrared spectroscopy combined with machine learning. *J. Food Compos. Anal.* **126**, 105871 (2024).

[24] Kusumiyati, K., Munawar, A. A. & Andasuryani, A. Investigating the possibility of using near infrared spectroscopy (NIRS) method in coffee beans caffeine and chlorogenic acid (CGA) determination by near infrared spectroscopy (NIRS). 797–809 (2025).

[25] Suhandy, D., Yulia, M., Widodo, S. & Naito, H. Al Riza, D. F. Fast authentication of Indonesian ground-roasted Arabica coffee adulterated with roasted soybean by portable LED-based fluorescence spectroscopy and chemometrics analysis. *Food Chem.* **479**, 143791 (2025). 40106917 10.1016/j.foodchem.2025.143791

[26] Baqueta, M. R., Marini, F., Rocha, R. B., Valderrama, P. & Pallone, J. A. L. Authentication and discrimination of new Brazilian Canephora coffees with geographical indication using a miniaturized near-infrared spectrometer. *Food Res. Int.* **172**, 113216 (2023). 37689959 10.1016/j.foodres.2023.113216

[27] Kusumiyati, K. & Putri, I. E. Comparison of color spectrophotometer and Vis/NIR spectroscopy on assessing natural pigments of cucumber applied with different ethephon concentrations. *Heliyon* **9**, (2023). 10.1016/j.heliyon.2023.e22564 PMC10730989 38125485

[28] Kusumiyati, K., Hadiwijaya, Y., Sutari, W. & Munawar, A. A. Global model for in-field monitoring of sugar content and color of melon pulp with comparative regression approach. *AIMS Agric. Food*. **7**, 312–325 (2022).

[29] Huang, W. et al. Identification of Alzheimer’s disease and vascular dementia based on a Deep Forest and near-infrared spectroscopy analysis method. *Spectrochim Acta Mol. Biomol. Spectrosc.* **326**, (2025). 10.1016/j.saa.2024.125209 39340951

[30] Deng, Z. et al. Detection of camellia oil adulteration based on near-infrared spectroscopy and smartphone combined with deep learning and multimodal fusion. *Food Chem.* **472**, (2025). 10.1016/j.foodchem.2025.142930 39826519

[31] Chen, Y. et al. Improvement of near-infrared spectroscopic assessment methods for the quality of Keemun black tea: utilizing transfer learning. *Food Res. Int.* **209**, (2025). 10.1016/j.foodres.2025.116184 40253124

[32] Kang, M. K., Hong, K. S., Yang, D. & Kim, H. K. Multi-scale neural networks classification of mild cognitive impairment using functional near-infrared spectroscopy. *Biocybern Biomed. Eng.* **45**, 11–22 (2025).

[33] Hadiwijaya, Y., Putri, I. E. & Kusumiyati, K. Water deficit-induced biochemical alterations and Vis/NIR spectral signatures in shallots. *Appl. Food Res.* **5**, 101532 (2025).

[34] Zhang, M. et al. Deep learning of the particulate and mineral-associated organic carbon fractions using a compositional transform and mid-infrared spectroscopy. *Geoderma* **455**, (2025).

[35] Munawar, A. A., Meilina, H. & Pawelzik, E. Near infrared spectroscopy as a fast and non-destructive technique for total acidity prediction of intact mango: comparison among regression approaches. *Comput. Electron. Agric.* **193**, 106657 (2022).

[36] Nyakuchena, M., Juntunen, C. & Sung, Y. Deep-learning-assisted near-infrared hyperspectral imaging for microplastic classification. *Powder Technol.* **457**, (2025).

[37] Ho, C. S. H. et al. Interpretable deep learning model for major depressive disorder assessment based on functional near-infrared spectroscopy. *Asian J. Psychiatr.* **92**, (2024). 10.1016/j.ajp.2023.103901 38183738

[38] Shi, Y. et al. Classification and rapid non-destructive quality evaluation of different processed products of Cyperus rotundus based on near-infrared spectroscopy combined with deep learning. *Talanta* **268**, (2024). 10.1016/j.talanta.2023.125266 37832457

[39] Sun, Y. et al. Attenuated total reflectance-flourier transformed infrared spectroscopy (ATR-FTIR) coupled with deep learning: a rapid method for geographical origin identification of sea cucumber Apostichopus japonicus. *Microchem. J.* **204**, (2024).

[40] Liu, L., Wang, B., Xu, X. & Xu, J. A tea classification method based on near infrared spectroscopy (NIRS) and transfer learning. *Infrared Phys. Technol.* **145**, (2025).

[41] Wang, K. et al. Experimental study on online detection of near-infrared spectroscopy suitable for continuous drug production. *J. Drug Deliv. Sci. Technol.* **104**, (2025).

[42] Li, C. et al. Simultaneous detection of citrus internal quality attributes using near-infrared spectroscopy and hyperspectral imaging with multi-task deep learning and instrumental transfer learning. *Food Chem.* **143996** 10.1016/J.FOODCHEM.2025.143996 (2025). 40168872

[43] Ding, F. et al. Non-invasive prediction of mango quality using near-infrared spectroscopy: assessment on spectral interferences of different packaging materials. *J. Food Eng.* **357**, 111653 (2023).

[44] Khatun, M. S., Masum, A., Al, Islam, M. H., Ashik-E-Rabbani, M. & Rahman, A. Short wave-near infrared spectroscopy for predicting soluble solid content in intact mango with variable selection algorithms and chemometric model. *J. Food Compos. Anal.* **136**, 106745 (2024).

[45] Munawar, A. A. & Wahyuni, D. Kusumiyati Near infrared spectroscopic data for rapid and simultaneous prediction of quality attributes in intact mango fruits. *Data Brief* **27**, (2019). 10.1016/j.dib.2019.104789 PMC6880090 31788517

[46] Song, Y. et al. A robust deep learning model for predicting green tea moisture content during fixation using near-infrared spectroscopy: integration of multi-scale feature fusion and attention mechanisms. *Food Res. Int.* **203**, (2025). 10.1016/j.foodres.2025.115874 40022390

[47] Hu, Y. et al. Comparison of machine learning and deep learning models for detecting quality components of vine tea using smartphone-based portable near-infrared device. *Food Control*. **174**, 111244 (2025).

[48] Ren, Y. et al. Estimation models for maize leaf water content at various stages using near-infrared spectroscopy. *Infrared Phys. Technol.* **145**, (2025).

[49] Ding, F. et al. Prediction of quality traits in packaged mango by NIR spectroscopy. *Food Res. Int.* **205**, 115963 (2025). 40032464 10.1016/j.foodres.2025.115963

[50] He, W., Huang, W., Popovic, T., Zhu, Z. & Zhang, X. Improving mango cold-damage and bruise detection using thermal imaging and flexible spectral sensing. *Food Control*. **172**, 111163 (2025).

[51] Wang, B. et al. A defect detection method for Akidzuki pears based on computer vision and deep learning. *Postharvest Biol. Technol.* **218**, 113157 (2024).

[52] Mei, M. & Li, J. An overview on optical non-destructive detection of bruises in fruit: technology, method, application, challenge and trend. *Comput. Electron. Agric.* **213**, 108195 (2023).

[53] Ansah, F. A., Amo-Boateng, M., Siabi, E. K. & Bordoh, P. K. Location of seed spoilage in mango fruit using X-ray imaging and convolutional neural networks. *Sci. Afr.* **20**, (2023).

[54] Al-Dairi, M. et al. Machine vision system combined with multiple regression for damage and quality detection of bananas during storage. *Appl. Food Res.* **4**, 100641 (2024).

[55] Nicolaï, B. et al. Nondestructive evaluation: detection of external and internal attributes frequently associated with quality and damage. *Postharvest Handl. Syst. Approach*. 10.1016/B978-0-12-822845-6.00014-2 (2021).

[56] Mishra, P. Chemometrics and intelligent laboratory systems a synergistic use of chemometrics and deep learning improved the predictive performance of near-infrared spectroscopy models for dry matter prediction in mango fruit. **212**, (2021).

[57] Mishra, P. & Woltering, E. El Harchioui, N. Improved prediction of ‘Kent’ mango firmness during ripening by near-infrared spectroscopy supported by interval partial least square regression. *Infrared Phys. Technol.* **110**, 103459 (2020).

[58] Gabriëls, S. H. E. J., Mishra, P., Mensink, M. G. J., Spoelstra, P. & Woltering, E. J. Non-destructive measurement of internal browning in mangoes using visible and near-infrared spectroscopy supported by artificial neural network analysis. *Postharvest Biol. Technol.* **166**, 111206 (2020).

[59] Olarewaju, O. O. et al. Model development for non-destructive determination of rind biochemical properties of ‘Marsh’ grapefruit using visible to near-infrared spectroscopy and chemometrics. *Spectrochim Acta Mol. Biomol. Spectrosc.* **209**, 62–69 (2019). 10.1016/j.saa.2018.10.027 30359850

[60] Hayati, R., Munawar, A. A. & Fachruddin, F. Enhanced near infrared spectral data to improve prediction accuracy in determining quality parameters of intact mango. *Data Brief.* **105571** 10.1016/j.dib.2020.105571 (2020). PMC7200245 32382601

[61] Marques, E. J. N., De Freitas, S. T., Pimentel, M. F. & Pasquini, C. Rapid and non-destructive determination of quality parameters in the ‘Tommy Atkins’ mango using a novel handheld near infrared spectrometer. *Food Chem.* **197**, 1207–1214 (2016). 26675859 10.1016/j.foodchem.2015.11.080

[62] Ong, P. et al. Sugarcane disease recognition through visible and near-infrared spectroscopy using deep learning assisted continuous wavelet transform-based spectrogram. *Spectrochim. Acta Mol. Biomol. Spectrosc* **324**, (2025). 10.1016/j.saa.2024.125001 39180971

[63] Posom, J. et al. Deep neural networks (DNNs) chemical compositions estimation of fresh durian in-line via near infrared spectroscopy. *J. Food Compos. Anal.* **142**, (2025).

[64] Li, X., Qiu, H., Ding, A. & Fan, P. Heavy metal concentrations prediction of marine sediments by visible-near infrared spectroscopy based on attention mechanism. *J. Hazard. Mater.* **484**, (2025). 10.1016/j.jhazmat.2024.136729 39631203

[65] Teerachaichayut, S. & Ho, H. T. Non-destructive prediction of total soluble solids, titratable acidity and maturity index of limes by near infrared hyperspectral imaging. *Postharvest Biol. Technol*. **133**, 20–25 (2017).

